# Commentary: A Metabolic Immune Checkpoint: Adenosine in Tumor Microenvironment

**DOI:** 10.3389/fimmu.2016.00332

**Published:** 2016-08-31

**Authors:** Peter Vaupel, Gabriele Multhoff

**Affiliations:** ^1^Department of Radiooncology and Radiotherapy, Klinikum rechts der Isar, Technische Universität München (TUM), Munich, Germany

**Keywords:** antitumor immunity, adenosine, VEGF, phosphatidylserine, radiotherapy

## Hypoxia Drives Malignant Progression

Hypoxia (i.e., critically reduced oxygen levels) is present in most human tumors (1). Systematic studies on the oxygenation status in the clinical setting have shown that the existence of hypoxic/anoxic subvolumes is a pathophysiological trait in solid malignancies with complex spatial and temporal heterogeneities, both within and between tumors of the same type. For many years, tumor hypoxia has been regarded as an obstacle for the control of tumors treated with standard radiotherapy (RT), some chemotherapies, and photodynamic therapy. During the last two decades, evidence is accumulating suggesting that hypoxia has a strong negative impact driving cancer cells toward a more aggressive phenotype, resulting from an increased mutagenicity (<0.1% O_2_, severe hypoxia), and hypoxia-driven regulation of a plethora of genes, promoting changes of the proteome and metabolome, preferentially through HIF-dependent mechanisms (<1% O_2_, modest-to-moderate hypoxia), ultimately leading to a poorer patient prognosis (2–4). In addition, hypoxia can enhance the expression of stem cell markers (5, 6) and can lead to a substantial inhibition of innate and adaptive antitumor immune responses [e.g., recently highlighted in Ref. (7)].

*Inter alia*, this latter aspect is addressed in a recent review by Ohta in this journal (8). Antitumor immune suppression – and thus tumor progression – can in part be directly mediated by hypoxia itself (adenosine-independent immune suppression) and, to a major part, be driven by HIF-dependent adenosine (ADO) production by immune and cancer cells with subsequent accumulation in the extracellular space (ECS), which contributes to a pro-cancer, hostile tumor microenvironment (9–11).

## Adenosine Counteracts Antitumor Immune Responses

**Figure 1:**
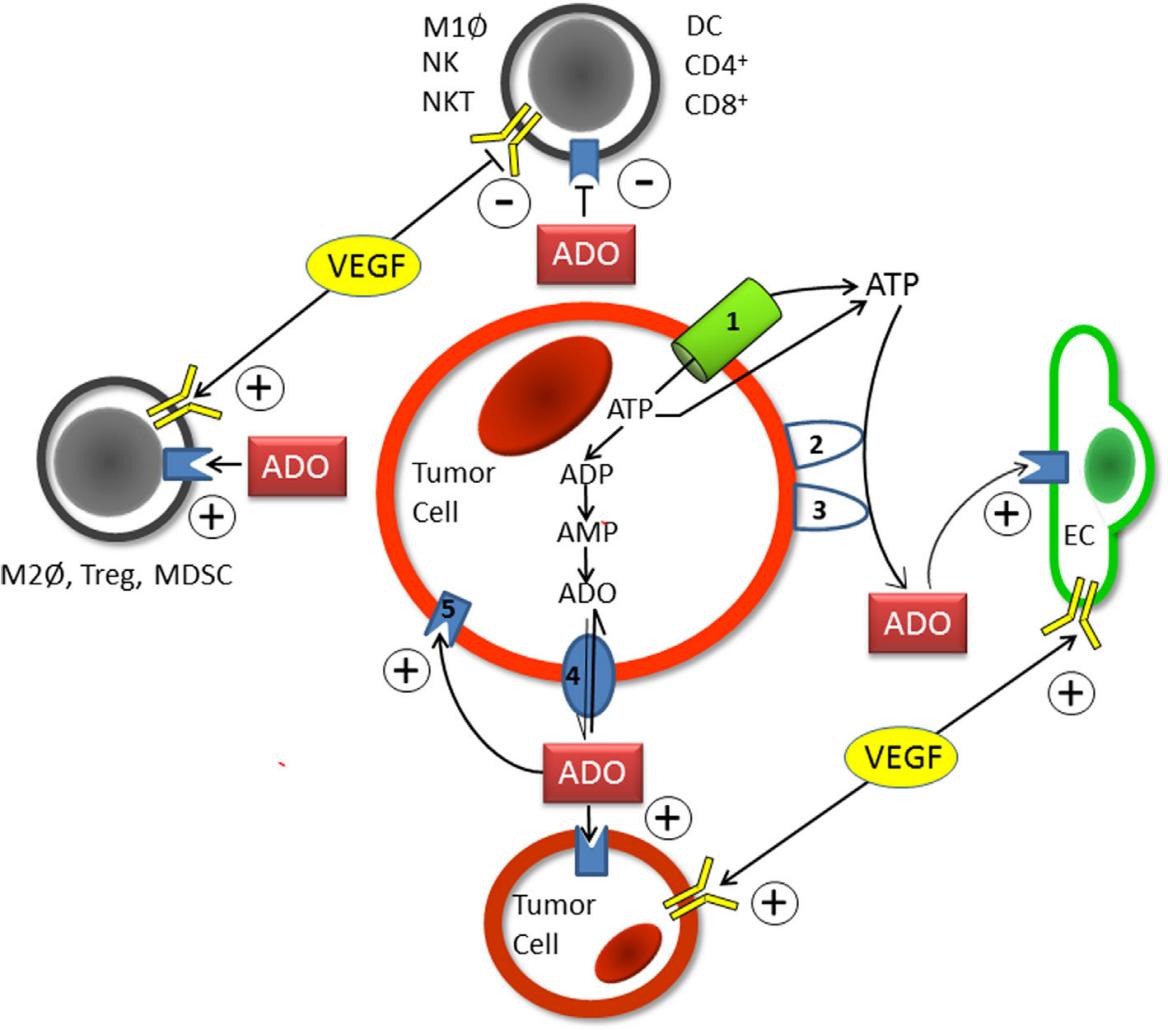
**Schematic diagram showing the individual steps of hypoxia-/HIF-1alpha-mediated adenosine (ADO) generation in the extracellular space (ECS) of tumor (and stromal) cells**. Upon hypoxic stress, ATP (ATP^4−^) is released into the positively charged ECS through pannexin-1 channels (1) or *via* exocytosis. Following the release of ATP into the ECS, the hypoxia-/HIF-1alpha dependent “tandem-enzymes” CD39 (2) and CD73 (3), the major nucleotide catabolizing enzymes, convert ATP into AMP and thereafter to ADO. Upon accumulation within the ECS and inhibition of ADO-uptake into the intracellular compartment by HIF-mediated inhibition of the nucleoside transporter ENT-1 (4), ADO acts in an autocrine and paracrine fashion in a sense that tumor-mediated immune suppression occurs (upper and left parts of Figure 1). Stimulating effects on endothelial (EC, right part of Figure 1) and tumor cells (lower part of Figure 1) are exerted through activation of A2A or A2B-receptors (5). Actions of VEGF/VEGFR expression on immune cells (and tumor and endothelial cells) are comparable to those elicited by ADO (see also Table S1A in Supplementary Material). Immune cells involved are specified in Table S1A in Supplementary Material. +, activation and stimulation; −, inhibition and suppression.

Adenosinergic effects on cancer and endothelial cells facilitating tumor progression and poor patient prognosis have been summarized in a recent review (9). Upon hypoxic stress, cancer cells release ATP^4−^ through PANX-1-channels and exocytosis into the ECS where nucleotides (ATP, ADP, and AMP) are converted into ADO by the HIF-sensitive, membrane-bound “tandem” ectoenzymes CD39/CD73. ADO actions are mediated mainly by HIF-sensitive A2A receptors on tumor and stromal cells of the tumor microenvironment (immune and endothelial cells included) using autocrine and paracrine pathways (Figure 1). A robust and long-lasting accumulation of ADO in the ECS is supported by a HIF-dependent inhibition of the nucleoside transporter ENT-1, which impedes a “downhill” ADO transport into the cell and thus a removal of ADO from the ECS. The rate of ADO removal from the ECS can further be reduced by HIF-dependent inhibition of the enzymes adenosine kinase (catalyzing AMP formation) and/or ADO-(ecto-)deaminase that favors inosine formation (9).

According to recent statements by Ohta [e.g., Ref. (8, 12)], the distinguished readership of this journal interested in this topic may get the impression that Blay et al. (13) were the first to detect and publish high intratumor ADO levels. Actually, in 1994, we studied the bioenergetic status of experimental tumors as a function of tumor size and oxygenation level (14, 15). In order to analyze the concentrations of different metabolites of ATP hydrolysis, ADO was assessed using HPLC techniques. A key result of these investigations was a very high ADO concentration in the range of 50–100 μM. ADO levels increased with enlarging tumor sizes and thus correlated with the extent of hypoxia (10, 15). In subsequent studies, “supraphysiologic” intratumor ADO contents in the micromolar range were confirmed (13). Extracellular ADO concentrations in normal tissues were found to be in the range of 10–100 nM [reviewed in Ref. (10)]. Our data published in 1994 clearly indicate that tumors – in contrast to normal tissues – accumulate ADO in concentrations high enough to even stimulate “low-affinity” A2A receptors.

In recent communications, we have emphasized that ADO can sabotage not only spontaneous antitumor immune responses but also antitumor immune functions artificially introduced with therapeutic intention, such as RT (9) and clinically achievable hyperthermia (HT) [see Table S1A in Supplementary Material (16)]. In addition, ADO can counteract immune therapies of solid tumors.

## VEGF and Phosphatidylserine as Immunosuppressive Signals in Tumors

Hypoxia-/HIF-driven expression of the vascular endothelial growth factor (VEGF) and activation of VEGFR also promote tumor evasion from immune responses [Figure 1 (17–20)]. Reversion of efficient antitumor immune responses may be a significant part of the benefits of antiangiogenic therapy (in addition to the debatable “normalization of the tumor vasculature” theory) using inhibitors targeting the VEGF/VEGFR pathway (17–20). Besides releasing an immunosuppressive and angiogenic secretome, accelerated tumor cell proliferation, growth promotion, increased invasion and metastasis, and development of chemoresistance have been observed upon autocrine activation of VEGF/VEGFR.

From these data, it is evident that ADO accumulation and increased VEGF/VEGFR expression are accomplices thwarting spontaneous antitumor immune responses (Figure 1). In addition, both hypoxia-/HIF-induced mechanisms can substantially attenuate antitumor immunity elicited by RT and HT (Table S1A in Supplementary Material).

Upon hypoxic stress, phosphatidylserine (PS) is frequently dysregulated in tumor cells and their microenvironment, thus antagonizing antitumor immunity [for a review, see Ref. (21)]. Although initially identified as an early signal of apoptosis, PS on the outer membrane leaflet on immature tumor endothelial cells (22), tumor exosomes (23), and viable tumor cells (24) provides a conserved immunosuppressive signal.

## Therapeutic Strategies Counteracting the Immunosuppressive Activities of Adenosine, Vascular Endothelial Growth Factor, and Phosphatidylserine

Measures to counteract immunosuppressive ADO actions have been discussed recently [(16), Table S1B in Supplementary Material]. These include respiratory hyperoxia, mild HT improving the oxygenation status of the tumor, antagonizing or downregulation of ADO receptors, inhibition of CD39 and CD73, co-blockade of immune checkpoint inhibitors CTLA-4 and PD-1/PDL-1, inhibition of the ENT-1 transporter or blockade of the ATP-release channel, HIF-pathway inhibition, enhancement of ADO degradation to inosine, and facilitation of AMP synthesis from ADO.

Blockade of the VEGF/VEGFR system by antiangiogenesis has been suggested to inhibit its deleterious effects on antitumor immune responses (Table S1B in Supplementary Material).

Reversal of the PS-induced antitumor immunosuppression can be stimulated by PS-targeting therapeutics [e.g., AnxA5, bavituximab, Table S1B in Supplementary Material (21)].

## Conclusion

Elevated ADO concentrations in the tumor microenvironment as a consequence of hypoxia/hypoxic stress were first described by Busse and Vaupel in 1994 (14, 15). This microenvironmental condition together with a hypoxia-/HIF-induced VEGF/VEGFR expression is sabotaging spontaneous and therapeutically triggered antitumor immune responses. Another signal compromising antitumor immunity is PS (25–31).

## Author Contributions

PV and GM equally contributed to the writing of this commentary article.

## Conflict of Interest Statement

The authors declare that the research was conducted in the absence of any commercial or financial relationships that could be construed as a potential conflict of interest.
