# Supplementary material for: Commentary: A Metabolic Immune Checkpoint: Adenosine in Tumor Microenvironment
Source: Front Immunol. 2016 Aug 31;7:332. doi: 10.3389/fimmu.2016.00332 (PMC5006596; doi:10.3389/fimmu.2016.00332)
Supplement: Supplementary file 1 [file Table_1.DOCX]

| Immune cells involved | Cellular function | Effector molecules | RT | HT | ADO | VEGF |
| --- | --- | --- | --- | --- | --- | --- |
| cDC, pDC | Antigen processing, antigen cross-presentation, T cell stimulation, maturation | IL-12,  IFN-α | **+** | **+** | **-** | **-** |
| Treg  (CD4^+^CD25^+^Foxp3^+^GARP^+^, LAP^+^) | Immunosuppression, tumor infiltration | IL-10,  TGF-β induces Treg expansion | **-** | **-** | **+** | **+** |
| MDSC (can differentiate to TAM) | Immunosuppression, promote tumor growth and invasion | TGF-β, VEGF, IL-6, NO, MMP, IL-10 | **-** | **-** | **+** | **+** |
| M1-macrophages | Tumor suppression,  Tumor cell killing | IFN-γ  TNF-α, IL-1, IL-6 | **+** | **+** | **-** | **-** |
| M2-macrophages,  TAM,  Tie-2^+^/CD16^+^ monocytes | Immunosuppression,  Tumor growth,  Tumor angiogenesis | IL-10, TGF-β  VEGF, bFGF, TNF, IL-1β, MMP, TNF  Ang-2 | **-** | **-** | **+** | **+** |
| NK cells,  NKT cells | Tumor cell killing | Perforin, granzyme, IFN-γ, Fas/Fas-L | **+** | **+** | **-** | **-** |
| CD4^+^  T helper 1 cells | Cytokine secretion, T and B cell help, activation of macrophages, inflammation | IFN-γ, TNF-α, IL-2, CD40-L, Fas/Fas-L | **+** | **+** | **-** | **-** |
| CD4^+^  T helper 2 cells | Cytokine secretion, T and B cell help | IL-4, IL-5, IL-9, IL-10, IL-13 | **+** | **+** | **-** | **-** |
| CD8^+^  cytotoxic T cells | Tumor cell killing | Perforin, granzyme, Fas/Fas-L | **+** | **+** | **-** | **-** |

Supplementary Table 1.

**A**. Immune cells, functions and effector molecules of the antitumor immune response and their modification by standard radiotherapy (RT), localized hyperthermia (HT), adenosine (ADO), and vascular endothelial growth factor (VEGF). +, activation, stimulation; -, inhibition, suppression

**B.** Therapeutic strategies counteracting the immunosuppressive activities of adenosine (ADO), vascular endothelial growth factor (VEGF), and phosphatidylserine (PS).

| Immunosuppressor | Strategies counteracting immunosuppressive activities (selection) | References |
| --- | --- | --- |
| ADO | Respiratory hyperoxia  Mild hyperthermia  Inhibition of ADO receptors  Inhibition of ectoenzymes CD39/CD73  Blocking of checkpoint inhibitors (i.e. PD-1/PDL-1, CTLA-4)  Inhibition of ENT-1 transporter  Inhibition of PANX-1 channels  Inhibition of HIF-regulated gene products  Increase in ADO degradation to inosine  Increase in AMP synthesis from ADO | reviewed in [9,25] |
| VEGF | Blocking of HIF activity  Targeting VEGF mRNA  Blocking of VEGF/VEGFR [by using antibody (bevacizumab), VEGF trap (aflibercept) or small molecules (sunitinib, sorafenib)]  Blocking of mTOR pathway with everolimus, temsirolimus  Combined blocking of VEGF/VEGFR and checkpoint inhibitors (e.g., PD-1/PDL-1, CTLA-4) | reviewed in [26,27,28,29,30,31] |
| PS | Blocking of PS with annexin A5 (Ca^2+^ dependent)  Blocking of PS with bavituximab  Blocking of export of PS-containing exosomes from tumor cells  Re-activation of PS-inhibited ADCC of NK cells | reviewed in [22] |

Abbreviations: Ab, antibody, ADCC, antibody dependent cellular cytotoxicity; AMP, adenosine monophosphate; Ang-2, angiopoietin-2; bFGF, basic fibroblast growth factor; c/pDC, conventional/plasmocytoid dendritic cells; ENT-1, equilibrative nucleoside transporter 1 (ADO transporter); Fas/Fas-L, Fas/Fas-ligand, CD95/CD95L; CTLA-4, cytotoxic T lymphocyte associated protein 4; GARP, glycoprotein A repetitions predominant, FoxP3, forkhead box P3; HIF, hypoxia inducible factor; INF, interferon, IL, interleukin, LAP, latency-associated peptide, MDSC, myeloid derived suppressor cell; MMP, matrix metalloproteinase; mTOR, mechanistic target of rapamycin; NK cell, natural killer cell; NKT, NK-like T cells; NO, nitric oxide; Panx-1; pannexin-1 (ATP channel); PD-1/PDL-1, programmed cell death-1/PD-1 ligand; TAM, tumor associated macrophage; TGF-β, tumor growth factor beta; TNF, tumor necrosis factor; Treg, regulatory T cells; VEGF, vascular endothelial growth factor; VEGFR, VEGF receptor.
